# Revolutionizing Cancer Treatment: Recent Advances in Immunotherapy

**DOI:** 10.3390/biomedicines12092158

**Published:** 2024-09-23

**Authors:** Rose Ghemrawi, Lama Abuamer, Sedra Kremesh, Ghadeer Hussien, Rahaf Ahmed, Walaa Mousa, Ghalia Khoder, Mostafa Khair

**Affiliations:** 1 College of Pharmacy, Al Ain University, Abu Dhabi P.O. Box 112612, United Arab Emirates; 2 AAU Health and Biomedical Research Center, Al Ain University, Abu Dhabi P.O. Box 112612, United Arab Emirates; 3 Department of Pharmaceutics and Pharmaceuticals Technology, College of Pharmacy, University of Sharjah, Sharjah P.O. Box 27272, United Arab Emirates; 4 Research Institute for Medical and Health Sciences, University of Sharjah, Sharjah P.O. Box 27272, United Arab Emirates; 5 Core Technology Platforms, New York University Abu Dhabi, Abu Dhabi P.O. Box 129188, United Arab Emirates

**Keywords:** cancer immunotherapy, monoclonal antibodies, CAR-T cell therapy, checkpoint inhibitors, cancer vaccines, personalized medicine

## Abstract

Cancer immunotherapy has emerged as a transformative approach in oncology, utilizing the body’s immune system to specifically target and destroy malignant cells. This review explores the scope and impact of various immunotherapeutic strategies, including monoclonal antibodies, chimeric antigen receptor (CAR)-T cell therapy, checkpoint inhibitors, cytokine therapy, and therapeutic vaccines. Monoclonal antibodies, such as Rituximab and Trastuzumab, have revolutionized treatment paradigms for lymphoma and breast cancer by offering targeted interventions that reduce off-target effects. CAR-T cell therapy presents a potentially curative option for refractory hematologic malignancies, although challenges remain in effectively treating solid tumors. Checkpoint inhibitors have redefined the management of cancers like melanoma and lung cancer; however, managing immune-related adverse events and ensuring durable responses are critical areas of focus. Cytokine therapy continues to play a vital role in modulating the immune response, with advancements in cytokine engineering improving specificity and reducing systemic toxicity. Therapeutic vaccines, particularly mRNA-based vaccines, represent a frontier in personalized cancer treatment, aiming to generate robust, long-lasting immune responses against tumor-specific antigens. Despite these advancements, the field faces significant challenges, including immune resistance, tumor heterogeneity, and the immunosuppressive tumor microenvironment. Future research should address these obstacles through emerging technologies, such as next-generation antibodies, Clustered Regularly Interspaced Short Palindromic Repeat (CRISPR)-based gene editing, and AI-driven drug discovery. By integrating these novel approaches, cancer immunotherapy holds the promise of offering more durable, less toxic, and highly personalized treatment options, ultimately improving patient outcomes and survival rates.

## 1. Introduction to Cancer Immunotherapy

Cancer immunotherapy is revolutionizing cancer treatment by harnessing the body’s immune system to identify and destroy malignant cells. This cutting-edge approach utilizes antibodies, immune cells, cytokines, and vaccines to specifically target cancer cells, offering a more precise and less toxic alternative to traditional therapies like chemotherapy [1].

Cytokines play a pivotal role in both innate and adaptive immune responses by facilitating communication between immune cells and mediating interactions within the tumor microenvironment (TME). They are essential in cancer immunotherapy due to their ability to expand and reactivate effector T and natural killer (NK) cells, enhance lymphocyte infiltration and persist in the TME, thereby overcoming resistance mechanisms. Cytokine therapy has been significant, with interferon-alpha (IFNα) approved for adjuvant therapy in high-risk melanoma and certain refractory cancers, and high-dose interleukin-2 (HDIL-2) approved for metastatic renal cell carcinoma and melanoma. However, challenges, such as dysregulation during malignant progression, rapid clearance, systemic toxicity, and complex biological properties, have limited their clinical use. Advances in cytokine biology, protein engineering, and biotechnology aim to improve pharmacokinetics, enhance tumor targeting, and reduce adverse effects, thereby increasing the potential of cytokine-based therapies for cancer treatment. Other cytokines like Granulocyte-Macrophage Colony-Stimulating Factor (GM-CSF), Interferon Gamma (IFNγ), Interleukin (IL)-7, IL-12, and IL-21 are under clinical investigation, reflecting ongoing efforts to develop more effective cytokine-based immunotherapies [2].

Immunotherapy strategies for cancer treatment are broadly categorized into passive and active approaches, each with distinct mechanisms and therapeutic goals. Passive immunotherapy involves administering pre-prepared immune components, such as monoclonal antibodies (mAbs) and immune cells, to provide immediate but temporary defense against cancer. These components are designed to target specific antigens in tumor cells. For example, mAbs like rituximab (targeting Cluster of Differentiation (CD)20 on malignant B lymphocytes) and trastuzumab (targeting Human Epidermal Growth Factor Receptor 2 (HER2) in breast cancer) work by mediating tumor cell lysis through mechanisms such as antibody-dependent cellular cytotoxicity (ADCC), complement-dependent cytotoxicity (CDC), and antibody-dependent cellular phagocytosis (ADCP). These mAbs offer rapid intervention by either inducing programmed cell death or blocking critical signaling pathways essential for tumor growth. Advances in antibody engineering, such as the development of chimeric or humanized mAbs, have significantly reduced immune responses against non-human components, thereby enhancing the efficacy and safety of mAbs in cancer therapy. While passive immunotherapy provides quick tumor control, particularly in aggressive or advanced-stage cancers, it often requires repeated administration due to its temporary nature [1,3].

Active immunotherapy, in contrast, works by engaging the patient’s own immune system to generate a sustained and long-lasting response. This approach stimulates the production of specific immune effectors, such as antibodies and T cells, which continue to combat the disease over time. Examples of active immunotherapy include cancer vaccines, which can generate tumor-specific T cell responses, and adoptive cell transfer (ACT), where autologous cells like tumor-infiltrating lymphocytes (TILs) or cytokine-induced killer (CIK) cells are expanded outside the body and then reintroduced to target and destroy cancer cells. Dendritic cell (DC)-based vaccines also fall into this category, in which DCs are used to present antigens and activate immune responses against tumors. Although active immunotherapy may take longer to show its effects, its goal is to provide a more durable and self-sustaining immune response, leading to longer-lasting protection against cancer recurrence. An example is HDIL-2, used in metastatic renal cell carcinoma and melanoma, which works by stimulating the body’s natural immune mechanisms to recognize and eliminate cancer cells [1,3].

Both passive and active immunotherapy strategies have demonstrated significant potential in disrupting biochemical pathways that are essential for tumor growth. They offer targeted treatments with fewer side effects compared to conventional therapies. The choice between these approaches depends on the specific clinical scenario, nature of the tumor, and desired therapeutic outcome. Ongoing research and clinical trials continue to explore ways to enhance the efficacy and safety of both passive and active strategies, such as combining mAbs with checkpoint inhibitors or integrating cytokine therapy with other treatment modalities [1,3].

## 2. Types and Mechanisms of Immunotherapy

The progression of cancer is influenced by the delicate balance between malignant cells and immune cells. Immunotherapies are designed to counteract the proliferation of cancer cells and shift this balance in favor of the immune system [4]. Numerous studies have highlighted the importance of modifying immune cells, such as T cells, and altering cancer cells to make them less aggressive [5]. To understand the roles of various immunotherapy types, we need to discuss the following:

(a) Monoclonal Antibodies: These synthetic proteins are designed to selectively attack cancer cells, representing a key example of personalized medicine in which drugs are tailored to each patient [6].
(b) Next-Generation Antibodies: Advances in antibody technology have led to the development of highly specific treatments that improve efficacy and reduce side effects.
(c) CAR-T Cell Therapy: T cells are extracted from a patient, genetically modified to better recognize cancer cells, and then reintroduced into the patient [7].
(d) Checkpoint Inhibitors: These agents act as brakes, allowing the body’s immune system to recognize and attack cancer cells more effectively [8].
(e) Cancer Vaccines: These vaccines introduce cancer antigens to the body, helping the immune system to recognize and attack cancer cells [9].
(f) Cytokine Therapy: Cytokines are proteins that stimulate immune cells to attack cancer cells. The inflammation caused by the immune system aids in the elimination of cancer cells [2].
(g) Immunomodulators: These agents enhance immunity against cancer cells by modifying the immune system to be more active [10].
(h) Oncolytic Viruses: These viruses, which can be either naturally occurring or synthesized in laboratories, are used to kill tumor cells [11].

## 3. Immunotherapy Agents and Approaches

### 3.1. Monoclonal Antibodies

The discovery of antibodies around 1890, credited to Paul Ehrlich, Emil von Behring, and Kitasato Shibasaburo, set the stage for a medical revolution. These small proteins have since become pivotal in treating a diverse range of diseases, including cancer. In the 1970s, Milstein and Köhler advanced antibody technology by pioneering the production of monoclonal antibodies in the laboratory. Their innovative “hybridoma” technique involved creating cell lines that secrete antibodies by fusing lymphocytes and myeloma cell lines [12]. The first human trial for mAb therapy targeting cancer was conducted in 1980 in a patient with lymphoma [6]. mAbs target specific antigens on tumor cells or those overexpressed by them, inducing tumor cell death through various mechanisms. One primary mechanism involves the inhibition of growth factor receptor signaling.

**Figure 1:**
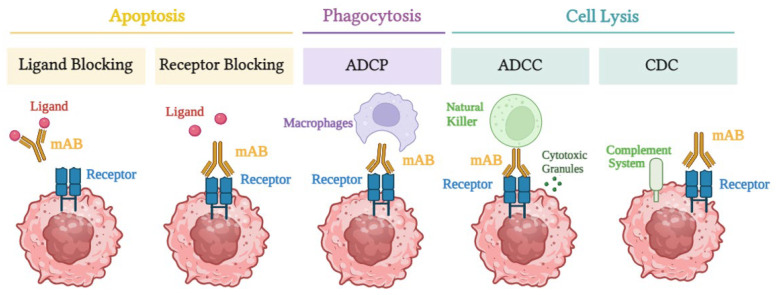
This figure illustrates the various mechanisms by which mAbs mediate their antitumor effects, including apoptosis, phagocytosis, and cell lysis. The Y-shaped structure (dark yellow) represents mAbs that can block ligands (pink balls) or receptors (blue rectangles) on cancer cells. Apoptosis: mAbs inhibit the binding of ligands to their receptors on cancer cells, thereby preventing pro-survival signaling and inducing cell death through either ligand blocking or receptor blocking. Phagocytosis (ADCP): mAbs coat cancer cells, allowing macrophages to recognize, engulf, and digest antibody-coated cells. Cell lysis: mAbs induce cell lysis through two main mechanisms: ADCC and CDC. In ADCC, NK cells bind to the antibody-coated cancer cells and release cytotoxic granules containing perforin and granzymes. Perforin forms pores in the cancer cell membrane, enabling granzymes to enter and induce apoptosis, ultimately leading to cell lysis. In CDC, the complement system is activated, which enhances the immune response by facilitating the elimination of pathogens or damaged cells. When mAbs bind to specific antigens on cancer cells, they initiate the complement cascade, a series of sequentially activated proteins that lead to the formation of the MAC. The MAC inserts into the cancer cell membrane, forming pores that disrupt the cell’s integrity, resulting in cell lysis. These combined mechanisms enhance the therapeutic efficacy of mAbs in targeting and destroying cancer cells.

When mAbs bind to their target receptors, they disrupt pro-tumor growth and survival signaling by altering receptor activation or obstructing ligand binding (Figure 1). Additionally, mAbs can activate the host immune system through mechanisms such as CDC, ADCP, and ADCC [13]. CDC is mediated by the complement system, a group of proteins that is activated when antibodies bind to the surface of a target cell. This binding triggers a proteolytic cascade, leading to the formation of a membrane attack complex (MAC), which creates pores in the target cell membrane, causing cell lysis and death. CDC is highly dependent on complement proteins and is particularly effective against cells with high expression of complement-activating antibodies on their surface [14]. In contrast, ADCC involves immune cells, primarily NK cells, that recognize the fragment crystallizable (Fc) region of antibodies bound to antigens on the target cell. Upon recognition, NK cells release cytotoxic granules containing perforin and granzymes, which induce apoptosis in the target cell. Unlike CDC, ADCC does not rely on the complement system but instead depends on the activity of immune effector cells [15]. Various immunoglobulins, including immunoglobulin G (IgG), immunoglobulin A (IgA), and immunoglobulin E (IgE), can facilitate ADCC, and multiple immune cells, including monocytes, macrophages, neutrophils, eosinophils, and dendritic cells, can also participate in this process [15].

Rituximab, the first U.S. Food and Drug Administration (FDA)-approved monoclonal antibody for treating hematological malignancies, is a genetically engineered chimeric antibody targeting CD20 [16]. Trastuzumab, the first humanized monoclonal antibody targeting HER2, has shown remarkable efficacy in treating HER2+ breast cancer by inhibiting cancer cell growth and promoting ADCC [17]. Bevacizumab, the first approved angiogenesis inhibitor, disrupts ligand-receptor interactions, impeding the pro-angiogenic pathway. Widely used as a Vascular Endothelial Growth Factor (VEGF) inhibitor, it is a key component in colorectal cancer treatment and is also used in various cancer types, such as cervical cancers [18].

Although mAbs have shown efficacy as solo treatments for certain patients, current approaches increasingly favor their use in combination with chemotherapy, radiation, and molecularly targeted drugs like tyrosine kinase inhibitors [19]. mAbs, such as Cetuximab, are designed to specifically target antigens on cancer cells, inhibiting growth factor receptor signaling, which is crucial for tumor survival and proliferation. By blocking these signals, mAbs disrupt tumor growth and can induce immune-mediated cell death through mechanisms such as ADCC or CDC.

On the other hand, chemotherapy is a more general approach that targets rapidly dividing cells, including cancer cells, by damaging their Deoxyribonucleic Acid (DNA) or disrupting their cellular machinery. While effective, chemotherapy often lacks specificity, leading to significant side effects due to its impact on healthy dividing cells. However, when combined with mAbs, chemotherapy can enhance the overall antitumor effect.

One of the key benefits of combining mAbs with chemotherapy is their potential to trigger immunogenic cell death (ICD). ICD is a form of cell death that not only kills tumor cells, but also stimulates the immune system to recognize and attack cancer cells more effectively. This combination can lead to increased infiltration of CD8+ T cells and NK cells into the tumor microenvironment, which is critical for sustained antitumor immune responses [20].

For example, in the treatment of head and neck squamous cell carcinoma (HNSCC), Cetuximab, which targets the Epidermal Growth Factor Receptor (EGFR), has been shown to enhance the effects of platinum-based chemotherapy. The combination improves overall survival and progression-free survival, as demonstrated in the EXTREME study, by not only inhibiting tumor growth directly, but also by enhancing the immune system’s ability to attack the cancer [21]. This synergistic approach leverages the strengths of both targeted therapy (through mAbs) and broad-spectrum cytotoxic therapy (through chemotherapy), leading to better patient outcomes.

Brentuximab vedotin, while tolerable as a solo therapy, is less effective as a frontline treatment for the elderly or comorbid patients with classical Hodgkin lymphoma; combining it with other agents shows promise for improving efficacy [22]. The FDA has approved two radio immunotherapies: yttrium-90 (90Y)-ibritumomab tiuxetan and iodine-131 (131I)-tositumomab. These therapies use mAbs targeting CD20 to deliver radioactive isotopes to lymphoma cells; however, they face various constraints [23].

Despite their success, antibody-based therapies face significant challenges, such as difficulties in crossing complex biological barriers within the body. As proteins, antibodies are susceptible to enzymatic and chemical degradation, leading to the rapid metabolism of certain antibody fragments. For example, blinatumomab has a brief half-life of approximately two hours, requiring continuous intravenous (IV) administration; the drug needs to be delivered through an IV over an extended period, often continuously for several days, to maintain its therapeutic effect. Moreover, mAbs can induce off-target cytotoxicity and adverse events such as cytokine release syndrome (CRS) and organ toxicity, posing life-threatening risks and limiting their widespread application. Additionally, the efficacy of many antibody-based immunotherapies is hindered by low response rates due to primary, adaptive, and acquired drug resistance mechanisms in cancers [8]. Although adverse reactions are more frequent in individuals who are new to immunotherapy, they generally remain rare. Notable concerns include hypersensitivity with potential adverse reactions such as angioedema, eczema, gastrointestinal complications, asthma, and conjunctivitis.

Recent advancements in the understanding of immune evasion mechanisms in cancer treatment, particularly those related to mAbs, have highlighted several strategies that tumors employ to circumvent immune surveillance. These mechanisms include the loss of tumor antigens, downregulation of antigen presentation, activation of immune checkpoint pathways, and induction of immune dysfunction and exhaustion.

Loss of Tumor Antigens: Tumor cells can undergo mutations or alternative splicing events that result in the loss or alteration of target antigens, making them less recognizable by mAbs. For instance, in B-cell malignancies, the loss of CD19 due to mutations or alternative splicing can lead to the production of truncated or non-functional proteins, thereby evading recognition by CAR-T cells [24]. This antigenic loss is a significant challenge in mAb-based therapies, as it contributes to tumor relapse and resistance [25,26].

Downregulation of Antigen Presentation: Tumor cells may downregulate the expression of major histocompatibility complex (MHC) molecules, which are essential for presenting antigens to T cells. This downregulation can occur through mutations in MHC genes or disruptions in antigen processing pathways, making it difficult for the immune system to detect and target these tumor cells. The loss of MHC-I can be partially compensated for by the activation of NK cells, which recognize the absence of MHC-I as a signal for cytotoxic action. However, tumors may counteract this by downregulating Natural Killer Group 2, member D (NKG2D) ligands, further evading NK cell-mediated killing [27,28].

Activation of Immune Checkpoint Pathways: Tumors often upregulate immune checkpoint proteins such as Programmed Death-Ligand 1 (PD-L1), which binds to Programmed Death-1 (PD-1) on T cells, leading to T cell exhaustion and reduced antitumor activity. This mechanism is particularly effective in creating an immunosuppressive environment that hampers the efficacy of mAbs and other immunotherapies [29].

### 3.2. Next-Generation Antibodies

With the advent of next-generation antibody technologies, including single-chain variable fragments, bispecific antibodies, Fc-engineered antibodies, nanobodies, and antibody-drug conjugates, healthcare and pharmaceutical sectors have become highly resourceful. These advancements have enabled the development of highly specific monoclonal antibody treatments for various diseases, such as cancer, autoimmune disorders, and infectious diseases [30].

(a) Bispecific Antibodies: These antibodies have been created to tackle drug resistance and enhance effectiveness. They can improve the efficacy and safety by concurrently identifying and binding to two distinct antigens or antigenic epitopes. Furthermore, they have the distinct advantage of guiding cytotoxic effector cells to the targeted antigen [31]. The first drug in this category to receive approval from the European Medicines Agency (EMA) is catumaxomab, specifically for the treatment of malignant ascites, as it targets both CD3 and Epithelial Cell Adhesion Molecule (EpCAM). In total, seven bispecific mAb agents are employed in cancer therapy [32].
(b) Fc-Engineered Antibodies: Fc receptors are proteins found on the surface of immune cells that attach to the Fc region of the antibodies. Fc engineering aims to enhance the effector functions or prolong the half-life of therapeutic antibodies by modifying their Fc regions. This modification can involve altering glycosylation to enhance interactions with Fc receptors or complement, or inducing mutations in the Fc region to boost the responses of CDC and ADCC [33]. Fc-engineered antibodies have significant clinical applications, particularly in cancer therapy, where they enhance the efficacy of monoclonal antibodies by improving ADCC and CDC [34,35,36,37]. These antibodies also have extended half-lives, making them effective for the long-term treatment of chronic diseases, including autoimmune disorders and infections [38,39,40,41]. By introducing specific mutations, such as glycosylation patterns, Fc-engineered antibodies improve interactions with Fc receptors, enhancing their therapeutic effects in cancer and infectious disease treatments [35,42,43]. Additionally, these antibodies can boost the immune system’s ability to fight tumors by blocking inhibitory signals from receptors like Fc gamma receptor IIB (FcγRIIB), enhancing their effectiveness in tumor therapy [44,45,46]. They are also used to neutralize autoreactive antibodies in autoimmune diseases, such as myasthenia gravis, by blocking their recycling [47,48,49]. Furthermore, bispecific antibodies generated through Fc engineering can simultaneously target multiple antigens, playing a critical role in tumor therapies by recruiting T cells to attack cancer cells [50,51,52].
(c) Antibody Fragments and Single-Domain Antibodies: Recombinant antibody fragments offer significant advantages over full-length antibodies. Their smaller size enhances tissue penetration for a more effective target reach, allowing them to bind epitopes in deeper antigen pockets [53]. These fragments are cost-effectively produced on a larger scale using bacterial or yeast systems. Additionally, they can be easily engineered to meet specific requirements. The fragment variable (Fv) fragment, which is the smallest functional unit of an antibody, consists solely of variable regions in the heavy or light chain. An FDA-approved therapeutic Fv fragment, Moxetumomab pasudotox-tdfk, is uniquely designed to target CD22-expressing cancer cells in hairy cell leukemia by combining an Fv fragment with a potent exotoxin [54].
(d) Nanobodies, also known as single-domain-based variable domains of heavy-chain antibodies (VHHs), are antibody fragments derived from the heavy-chain-only IgG antibodies found in the Camelidae family. Due to their small size, simple structure, high antigen-binding affinity, and remarkable stability under extreme conditions, nanobodies have the potential to overcome several limitations of conventional monoclonal antibodies. For many years, nanobodies have been of great interest in various research fields, particularly in the diagnosis and treatment of diseases. This interest culminated in the approval of the world’s first nanobody-based drug, Caplacizumab, in 2018 [55].

Nanobodies, due to their unique properties, such as small size, excellent stability, low immunogenicity, and ability to recognize hidden epitopes, have shown significant clinical relevance in disease diagnosis and treatment. In cancer diagnosis, nanobodies can detect biomarkers like Cancer Antigen 125 (CA125), Prostate-Specific Antigen (PSA), and Alpha-Fetoprotein (AFP) with high sensitivity, enabling early screening, diagnosis, and monitoring of disease progression [56,57,58]. Nanobodies are also effective as probes in noninvasive imaging techniques, such as Positron Emission Tomography/Computed Tomography (PET/CT), providing clear images of tumors and metastatic lesions, which can significantly enhance the detection and monitoring of cancers like breast cancer, melanoma, and non-small-cell lung cancer (NSCLC) [59,60,61]. In infectious diseases, nanobodies have been employed to detect pathogens like Severe Acute Respiratory Syndrome Coronavirus-2 (SARS-CoV-2), Human Immunodeficiency Virus Type 1 (HIV-1), and various bacterial infections with high specificity and sensitivity, making them valuable tools for early and accurate diagnosis [62,63,64]. Their ability to penetrate tissues and bind to specific antigens also makes them suitable for detecting chronic conditions like Alzheimer’s disease, cardiovascular diseases, and neurodegenerative disorders, where they can be used to visualize and quantify biomarkers in vivo [65,66,67,68]. Moreover, the application of nanobodies extends to the detection of small molecules and toxins, where they exhibit high stability under harsh conditions and sensitivity for detecting contaminants in food and the environment [69,70,71]. These characteristics make nanobodies a promising alternative to conventional antibodies for both diagnostic and therapeutic applications.

(e) Antibody-Drug Conjugates (ADCs):

ADCs represent a cutting-edge treatment modality that combines monoclonal antibodies with cytotoxic agents, facilitating the precise delivery of powerful drugs to cancer cells expressing specific surface antigens. This targeted approach is designed to enhance therapeutic effectiveness while minimizing systemic toxicity, thereby differentiating ADCs from conventional chemotherapy [72].

The development and approval processes for ADCs involve rigorous clinical trials and evaluations to ensure their efficacy and safety. Currently, numerous ADCs are in various stages of clinical trials, and 15 have successfully received approval from major regulatory bodies, including the FDA and EMA, for the treatment of both hematologic malignancies and solid tumors. Over the past 23 years, ADCs such as Gemtuzumab ozogamicin (Mylotarg^®^), Brentuximab vedotin (Adcetris^®^), Inotuzumab ozogamicin (Besponsa^®^), Polatuzumab vedotin (Polivy^®^), Belantamab mafodotin (Blenrep^®^), Loncastuximab tesirine (Zynlonta^®^), and Moxetumomab pasudotox (Lumoxiti^®^) have been specifically developed for hematologic cancers, with Lumoxiti^®^ using an immunotoxin rather than a chemotherapeutic agent as its payload.

For solid tumors, approved ADCs include Ado-trastuzumab emtansine (Kadcyla^®^), Fam-trastuzumab deruxtecan (Enhertu^®^), Enfortumab vedotin (Padcev^®^), Sacituzumab govitecan (Trodelvy^®^), Tisotumab vedotin-tftv (Tivdak^®^), Mirvetuximab soravtansine (ELAHERE^®^), Disitamab vedotin (Aidixi^®^), and Cetuximab sarotalocan (Akalux^®^) [73].

### 3.3. CAR-T Cell Therapy 

**Figure 2:**
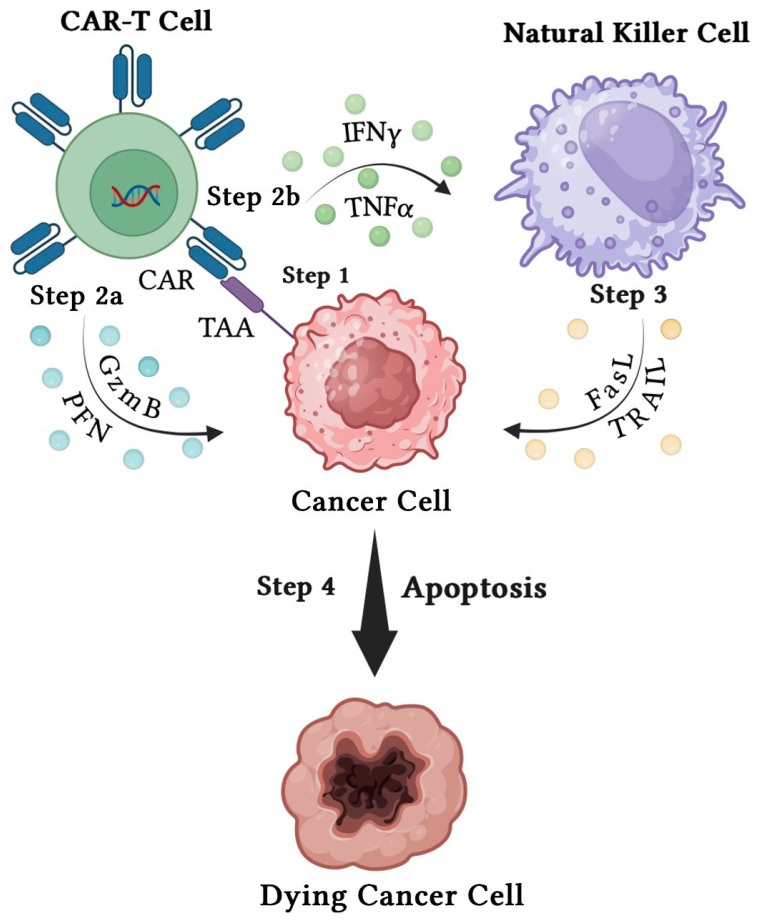
This figure illustrates the mechanism by which CAR-T cell therapy targets cancer cells. Step 1: CAR-T cells engineered to express CARs specifically recognize TAAs on the surface of cancer cells. Step 2a: Upon TAA recognition, CAR-T cells initiate an antitumor response by releasing cytolytic molecules, such as Granzyme B (GzmB) and Perforin (PFN), which directly induce apoptosis in the cancer cell. Step 2b: Simultaneously, CAR-T cells secrete pro-inflammatory cytokines, including IFNγ and tumor necrosis factor-alpha (TNFα), which activate NK cells. Step 3: Activated NK cells engage in antitumor activity by releasing their own cytolytic molecules, such as Fas Ligand (FasL) and Tumor Necrosis Factor-related Apoptosis-Inducing Ligand (TRAIL), which further potentiate the killing of cancer cells. Step 4: The combined action of CAR-T cells and NK cells results in cancer cell apoptosis, as depicted by the dying cancer cell.

CAR-T cells (Figure 2) primarily function by identifying specific surface antigens on tumor cells, without requiring antigen processing and presentation. This means that CAR-T cells recognize antigens independently of MHC restrictions. The structure of CARs includes an extracellular antigen recognition domain, a transmembrane domain, and an intracellular signaling domain. The extracellular domain, known as the single-chain variable fragment (scFv), precisely recognizes surface antigens on tumor cells. Tumor antigens typically fall into two categories, tumor-associated antigens (TAAs) and tumor-specific antigens (TSAs), with most being TAAs. TAAs are antigens found on tumor cells and some normal cells, but they are expressed at higher levels in tumors. In contrast, TSAs are antigens only found on tumor cells, making them more specific targets for cancer therapies. Once the scFv identifies TAAs, CAR-T cells are activated, and transmit signals to the intracellular domain [74].

Eshhar et al. initially characterized CAR-T cells by combining a murine scFv with a CD3 signaling chain, which was subsequently introduced into human T cells [75]. The first-generation CAR design included an scFv antigen recognition domain and an intracellular CD3ζ activation domain. However, due to the absence of costimulatory signals, these first-generation CARs exhibited limited proliferative capacity and antitumor effects. The second-generation CAR design addressed this limitation by incorporating an additional costimulatory domain, such as CD28, Tumor Necrosis Factor Receptor Superfamily Member 9 (4-1BB), Tumor Necrosis Factor Receptor Superfamily Member 4 (OX40), Inducible T-cell Costimulator (ICOS), enhancing their proliferative capacity and cytokine release, thus improving antitumor effects. Currently, commercially available CAR-T cell products predominantly use the second-generation CAR design.

The third-generation CAR design involves two distinct costimulatory molecules, such as CD28 and 4-1BB [74]. The fourth-generation CAR construct, also known as “armored CAR” or T cells Redirected for Universal Cytokine Killing (TRUCK), not only targets and kills cancer cells, but also secretes cytokines in the tumor microenvironment, enhancing the immune response. TRUCKs undergo additional modifications to secrete cytokines or express suicide genes such as IL-7, IL-12, IL-15, IL-21, and Inducible Caspase-9 (iCaspase-9). These modifications enhance the potential of fourth-generation CAR-T cells to eliminate tumor cells by activating endogenous immune responses [76,77].

IL-7 and IL-15 are essential for promoting the survival, proliferation, and persistence of CAR-T cells in a patient’s body. These cytokines help maintain the long-term activity of CAR-T cells, which is crucial for sustained tumor control, especially in solid tumors where T-cell persistence is a significant challenge [78,79]. IL-12 has been engineered into some CAR-T cells to enhance their ability to reshape the tumor microenvironment, making it more conducive to T-cell activity. IL-12 can promote the production of pro-inflammatory cytokines and enhance the cytotoxic activity of CAR-T cells, which is critical for overcoming the immunosuppressive environment often found in solid tumors [80]. IL-21 further augments the expansion of CAR-T cells and supports their differentiation into memory T cells, which are associated with long-term immune surveillance against tumor recurrence [79]. Additionally, the inclusion of iCaspase-9 serves as a safety switch in advanced CAR-T cells. If severe adverse effects such as CRS occur, iCaspase-9 can be activated to induce apoptosis in CAR-T cells, thereby controlling the therapy’s potential toxicity and improving patient safety [81,82].

Despite their potential, much remains unknown about the characteristics of fourth-generation CAR-T cells. Numerous studies have shown that relying solely on CAR-T cell therapy has limited effectiveness in treating solid tumors [83,84]. Consequently, researchers have explored innovative combinations to enhance the synergistic potential of CAR-T cell therapy.

When administered at low doses, chemotherapy can play an immunomodulatory role by stimulating dendritic cell activation and tumor antigen presentation to CAR-T cells [85]. Simultaneously, it hampers the suppressive immune cells, thereby enhancing the persistence of CAR-T cells. Additionally, low-dose chemotherapy sensitizes tumor cells to CAR-T cell activity by facilitating the penetration of granzyme B into the tumor cells [84,86,87]. The combined therapy effectively tackles the challenge of CAR-T cell trafficking to the TME, leading to more robust tumoricidal responses and elevated rates of tumor rejection [88]. These beneficial outcomes are attributed to oxaliplatin-induced secretion of chemokines by tumor-associated macrophages, attracting T cells, and enhancing CAR-T cell infiltration, thereby remodeling the TME to make tumors more sensitive to anti-PD-L1 treatment [89].

Radiotherapy can directly eliminate cancer cells through apoptosis and necrosis, inducing the maturation and activation of dendritic cells, which facilitates tumor antigen presentation [90]. Following radiation, the release of damage-associated molecular patterns (DAMPs) and IFNγ occurs, promoting the migration and infiltration of CAR-T cells into the tumor [91]. The combination of CAR-T cell therapy and radiotherapy results in a synergistic antitumor effect [92,93].

An alternative strategy involves combining CAR-T cells with oncolytic viruses to address significant challenges that limit the efficacy of CAR-T cell therapy alone. The virus can penetrate tumor cells, which is a challenging task for CAR-T cell monotherapy. Additionally, the oncolytic virus contributes to tumor debulking by dismantling the molecular shield used by certain solid tumors to evade immune system attacks, enhancing CAR-T cell infiltration into the tumor site. Moreover, the oncolytic virus transforms the immunosuppressive TME into a pro-inflammatory setting, leading to increased proliferation and survival of CAR-T cells [94,95,96]. Numerous studies have demonstrated that this approach enhances antitumor efficacy against solid tumors [97,98].

Localized ablative therapies such as microwave ablation eliminate tumors by inducing hyperthermic damage in cancer cells. These therapies also prompt the release of immunomodulatory factors, including danger signals, tumor antigens, and cytokines, initiating an antitumor immune response [99]. In a recent study, the combination of microwave ablation with CAR-T cells targeting the receptor tyrosine kinase AXL in patient-derived xenografts from non-small cell lung cancer demonstrated improvements in infiltration, activation, persistence, and tumor-killing efficacy [100]. Moreover, the photothermal ablation of tumors, when combined with CAR-T cells specific to chondroitin sulfate proteoglycan-4, exhibited superior antitumor activity against the melanoma cell line WM115 [101].

Significant obstacles facing CAR-T cell therapy in the context of solid tumors include the identification of genuinely specific tumor antigens, addressing issues related to tumor antigen evasion, and improving the trafficking, infiltration, expansion, persistence, and functionality of CAR-T cells within the challenging TME. To overcome these challenges and enhance the effectiveness of CAR-T cells in treating solid tumors, diverse strategies have been devised. These include optimizing CAR constructs and identifying innovative therapeutic combinations, thereby augmenting the specificity, infiltration, and efficacy of CAR-T cell treatment while modulating inhibitory conditions [97].

CAR-T cell therapy has led to significant advancements in the treatment of certain solid tumors. Interleukin-13 Receptor Alpha 2 (IL-13Rα2) is highly expressed in glioblastoma tumor cells but is seldom found in normal brain cells, making it a compelling target for CAR-T cell therapy in glioblastoma cancer. In a study by Brown and colleagues (National Clinical Trial (NCT)02208362), multi-dose treatment with IL-13Rα2-CAR-T cells resulted in complete tumor regression for nearly eight months in a patient with disseminated glioblastoma [102]. Another clinical trial (NCT00730613) targeting the same tumor antigen used anti-IL-13Rα2-CAR-T cells to treat three patients with recurrent glioblastoma. The therapy was well tolerated, with controlled brain inflammation observed in all the patients with recurrent disease. Although one patient experienced brief remission, it may have been attributed to the loss of the IL-13Rα2 antigen on the relapsing tumor [103].

Promising results were observed in a phase I/II clinical trial (NCT00902044) involving HER2-CAR-T cells for the treatment of 19 patients with HER2-positive sarcomas, including osteosarcomas, primitive neuroectodermal tumors, Ewing sarcoma, and protofibroblastic small round cell tumors. Among the 17 evaluable patients, four showed stable disease for three to 14 months, and three of them received no additional therapy and underwent successful tumor removal, with one displaying over 90% tumor necrosis [104].

### 3.4. Immune Checkpoint Inhibitors 

Immune checkpoint inhibitors (ICIs) represent a highly promising category of cancer immunotherapy, with multiple drugs receiving FDA approval for more than nine cancer types over the past decade [105]. ICI therapy operates on the principle that T cells possess evolutionarily conserved inhibitory signals, acting as “checkpoints” to control activation [106]. Shortly after activation, T cells elevate the expression of inhibitory receptors cytotoxic T lymphocyte antigen 4 (CTLA-4) and PD-1. These receptors then bind to the costimulatory ligands CD80, CD86, PD-L1, and Programmed Death-Ligand 2 (PD-L2), which are expressed by tumor cells, regulatory T cells (Tregs), myeloid cells, and antigen-presenting cells (APCs). This binding dampens the activation of cytotoxic T cells, leading to immune suppression and tumor growth [107,108,109,110]. ICIs alleviate this inhibition, enabling primed and activated cytotoxic T cells to target and destroy cancer cells [111,112]. ICI therapy has proven successful in various challenging cancers [113].

(a) PD-1 Inhibitors: PD-1 functions as an inhibitory receptor, playing a crucial role in regulating programmed death signaling to modulate T-cell-mediated responses [114]. Engagement with PD-1 can diminish cytokine secretion, such as IL-2, IFN-γ, and TNF-α, as well as cell proliferation, by interfering with the CD28-costimulatory signaling pathway. The expression of PD-1 has been identified in various immune cell types within the TME, including activated monocytes, DCs, NK cells, T cells, and B cells [115]. Immunotherapies targeting the PD-1 pathway have transformed the treatment landscape of various cancers, including Merkel cell carcinoma (MCC), melanoma, HNSCC, and NSCLC [116].

PD-1 inhibitors function by blocking the interaction between the PD-1 receptor on T cells and its ligands PD-L1 and PD-L2, which are often expressed in tumor cells and antigen-presenting cells [117]. Normally, this interaction sends an inhibitory signal to T cells, reducing their activity, proliferation, and cytokine production as part of the body’s mechanism to prevent autoimmunity and to maintain immune balance. Tumor cells exploit this pathway to evade immune detection by “turning off” T cells. PD-1 inhibitors such as Pembrolizumab and nivolumab disrupt this immune checkpoint, thereby reactivating T cells and enhancing their ability to recognize and attack tumor cells. This reactivation also leads to increased cytokine production, improved cytotoxic activity, and a shift in the tumor microenvironment toward a more pro-inflammatory state, further supporting the immune response against the tumor. Thus, PD-1 inhibitors play a critical role in reversing immune suppression and restoring effective immune regulation in the context of cancer [117].

(b) PD-L1 Inhibitors: PD-L1 and PD-L2 serve as the two ligands for PD-1 [118]. Both tumor and immune cells have the capability to express PD-L1, making it a valuable biomarker for predicting responses to anti-PD-1/PD-L1 antibodies in certain patients with various types of cancer [119]. Also recognized as CD274, PD-L1 contributes to inhibiting the cancer immunity cycle by binding to negative regulators of T-cell activation, such as PD-1 and CD80 [120]. Consequently, the ligation of PD-L1 is known to inhibit the migration and proliferation of T cells, thereby constraining the killing of tumor cells [121]. The US FDA has granted approval for three PD-L1 inhibitors, namely Atezolizumab, Durvalumab, and Avelumab, which have been used to treat some solid tumors, including NSCLC, HNSCC, melanoma, and MCC [116].
(c) CTLA-4 Inhibitor: CTLA-4, a protein within the immunoglobulin superfamily, was first identified in a cytotoxic T lymphocyte (Complementary DNA (cDNA) library as being primarily expressed by activated T cells [122]. CTLA-4 is crucial for modulating T-cell activation, particularly during the early phases. Its primary function is to inhibit the activity of CD28, a costimulatory receptor on T cells [123]. Although both CTLA-4 and its homolog, CD28, bind to the same ligand, B7, on B cells and APCs, CTLA-4 stimulation results in T cell-mediated suppression of antibody formation and prevents allograft rejection [124,125].

When CTLA-4 binds to B7 molecules, it transmits an inhibitory signal to the T cell, reducing T cell proliferation and cytokine production. This mechanism is vital for maintaining immune homeostasis and preventing autoimmunity by ensuring that T cells do not overreact with antigens, including self-antigens. CTLA-4 inhibitors, such as ipilimumab, block the interaction between CTLA-4 and B7 molecules, thereby enhancing T-cell activation and proliferation. By preventing CTLA-4 from delivering its inhibitory signals, these inhibitors effectively unleash the immune system, allowing T cells to attack tumor cells more vigorously. The blockade of CTLA-4 has been shown to promote the expansion of effector T cells and reduce the number of Tregs in the tumor microenvironment, further amplifying the antitumor immune response [126].

In 1994, it was discovered that the expression kinetics of CTLA-4 significantly differ from those of CD28. CTLA-4 expression increases for 2–3 days after T Cell Receptor (TCR)/CD3-mediated T cell activation, starting approximately 24 h after TCR triggering, while CD28 is expressed on naive T cells. These observations imply that CTLA-4 plays a crucial role in regulating activated T cells and that the absence of CTLA-4 results in uncontrolled T cell proliferation. This new understanding of CTLA-4’s mode of action prompted researchers to investigate whether blocking CTLA-4 could enhance antitumor immune responses [127].

The relationship between chemotherapy and the immune system is complex and bidirectional. The effectiveness of chemotherapy often depends on the functional immune system, and chemotherapy can influence the immunogenicity of tumors. In states of immune deficiency, the effectiveness of various chemotherapeutic agents is diminished. Moreover, traditional chemotherapy can boost the immunogenic characteristics of cancer cells, thereby triggering immune effector cells. Chemotherapy can increase antigen release and enhance immunogenicity, potentially increasing vulnerability of the immune system [128].

For example, the integration of Pembrolizumab, an anti-PD-1 antibody, with carboplatin and paclitaxel has been shown to reduce the mortality risk in individuals with advanced NSCLC. Similarly, combining Atezolizumab, an anti-PD-L1 antibody, with carboplatin and paclitaxel has demonstrated enhanced treatment efficacy for advanced NSCLC [129].

There is substantial evidence indicating that ICIs, particularly PD-1 blocking antibodies, can rejuvenate the antitumor functions of cytotoxic T lymphocytes [81]. The combination of CARs and ICIs has yielded promising results. Notably, PD-1 blocking antibodies secreted by CAR-T cells can competitively bind to PD-1, thereby enhancing the proliferation and cytotoxicity of CAR-T cells [130,131,132,133].

Despite the promising outcomes of ICIs in adults, there is limited information regarding their safety in children. Studies have shown that dose-dependent adverse events associated with CTLA-4 blockade, ranging from mild to moderate, occur in over 70% of patients. Additionally, a meta-analysis of 18 clinical trials indicated an elevated risk of treatment-related mortality (TRM) in patients receiving higher doses (10 mg/kg) of CTLA-4 inhibitors [84]. The toxicities associated with PD-1/PD-L1 blockade were comparatively less severe than those associated with CTLA-4 inhibitors. Fatigue has emerged as the most common adverse event, affecting 16–37% of patients receiving PD-1 inhibitors and 12–24% of those receiving PD-L1 inhibitors [134].

It is particularly concerning that unpredictable off-target effects on vital organs can pose a life-threatening risk to children, whose organs are less mature, potentially leading to lifelong disabilities. Moreover, the TME, which is composed of cancer and immune cells, adds complexity to the treatment process [134].

Over the past few years, numerous clinical trials have been conducted using PD-1/PD-L1 ICIs, and their therapeutic outcomes have been assessed from various angles, such as overall survival (OS), objective response rate (ORR), pathologic complete response (pCR) rates, disease-free survival (DFS), and median progression-free survival (PFS).

The German phase II GeparNUEVO trial investigated the addition of durvalumab to standard neoadjuvant chemotherapy in 174 patients with triple-negative breast cancer. Despite a numerical increase in pCR rates with durvalumab, statistical significance was not reached. However, patients who received durvalumab before chemotherapy showed better outcomes, with a more significant increase in pCR. A subsequent report with 42 months of follow-up revealed an improved 3-year invasive DFS, reduced distant recurrence risk, and increased 3-year OS in the combination group. Although this was a small study, these promising results have sparked further investigation of ICIs in this setting [135,136].

The combined blockade of CTLA-4 and PD-1 is supported by a robust immunological rationale and has been established as a standard option in certain cancers like kidney cancer or melanoma [137,138,139].

In the case of advanced esophagogastric cancer, the CHECKMATE-032 trial examined the nivolumab/ipilimumab combination at various doses and schedules in 160 patients [140]. Despite the increased toxicity of the anti-CTLA-4 component, the observed activity justified further evaluation in the subsequent phase III CHECKMATE-649 study [141]. Another combination, involving tremelimumab (a fully humanized anti-CTLA-4 antibody) and durvalumab, was assessed in a randomized phase II study of advanced gastric/gastroesophageal junction (GEJ) cancers. The 12-month OS rates for these combined immunotherapies were comparable to those observed with ipilimumab and nivolumab [142].

#### Recent Advancements in CAR-T Cell Combination Therapy with Checkpoint Inhibition

Checkpoint signaling regulates immune responses by downregulating excessive activity to prevent autoimmunity. Tumor cells exploit this pathway to evade immune detection, leading to the exhaustion of immune cells. Checkpoint blockade (CPB) has emerged as a key cancer immunotherapy strategy to enhance immune function and increase immune cell persistence. Combining CAR-T cell therapy with CPB, particularly PD-1 blockade, has shown promising results in improving CAR-T cell effectiveness [143].

Studies have demonstrated that combining CAR-T cells with PD-1 inhibitors like Pembrolizumab restores key immune functions and enhances CAR-T cell persistence in treating cancers such as metastatic melanoma. However, systemic PD-1 blockade raises concerns, including suboptimal targeting in the tumor microenvironment and significant side effects, such as renal failure and pancreatitis [144,145]. To mitigate these issues, targeted CPB delivery via CAR-T cells is being explored, with preconditioning of CAR-T cells in specific cytokine environments (e.g., IL-7/IL-15) showing improved outcomes and reduced side effects [146].

Further advancements involve using dominant-negative receptors (DNR) and short hairpin RNA (shRNA) to enhance CAR-T cell function. CAR-T cells engineered with PD-1 DNR or PD-1–targeting shRNA show increased persistence, cytokine production, and antitumor activity by effectively neutralizing PD-1-mediated immunosuppression. Additionally, knocking down adenosine 2A receptors (A2ARs) and CTLA-4 in CAR-T cells has significantly boosted their function and expansion [147,148].

However, this approach also presents significant clinical challenges and potential adverse effects that need to be carefully managed. Combining CTLA-4 and PD-1 inhibitors significantly increases the risk of immune-related adverse events (irAEs). These toxicities arise because an enhanced immune response can also target healthy tissues, leading to autoimmune-like conditions. Common irAEs include colitis, dermatitis, hepatitis, and endocrinopathies, with more severe cases involving neurotoxicity and cardiotoxicity [149,150].

Managing the toxicities associated with combined CTLA-4 and PD-1 blockade involves the use of immunosuppressive agents like corticosteroids, which have proven effective in controlling irAEs. However, the use of steroids can dampen the antitumor immune response, creating a delicate balance between managing adverse effects and maintaining therapeutic efficacy. There is a need for global management guidelines to standardize the treatment of irAEs, ensuring that patients receive appropriate care while minimizing the impact on the effectiveness of cancer therapy [149].

In conclusion, although the combination of CTLA-4 and PD-1 inhibitors offers a powerful tool in cancer immunotherapy, it also presents significant challenges that require careful consideration and management. The balance between enhancing antitumor immunity and controlling immune-related toxicities will be critical for maximizing the clinical benefits of this therapeutic approach.

### 3.5. Cancer Vaccines 

The development of cancer vaccines began in the 1980s with the pioneering development of tumor lysate-based vaccines targeting colorectal cancer [151]. By 1990, a groundbreaking vaccine synthesized from a human tumor antigen had emerged, marking a significant milestone in cancer immunotherapy [152]. This momentum continued in 2010 when the FDA approved Sipuleucel-T, the first-ever therapy for metastatic castrate-resistant prostate cancer, heralding a new era of targeted treatment [153].

In 1996, the field took another leap forward with clinical trials testing a first messenger Ribonucleic Acid (mRNA)-based cancer vaccine, where dendritic cells were pulsed with mRNA [154]. The success of the Pfizer-BioNTech SARS-CoV-2 mRNA vaccine in 2021 has reignited interest in mRNA-based cancer vaccines, spurring a new wave of clinical trials and innovative therapies aimed at harnessing the power of mRNA technology to fight cancer [155]. Current clinical trials are exploring mRNA-based cancer vaccines targeting various cancers such as NSCLC, colorectal cancer, gastroesophageal adenocarcinoma, urothelial carcinoma, melanoma, unresectable solid tumors, bladder cancer, triple-negative breast cancer, renal cancer, head and neck cancer, advanced esophageal cancer, postoperative hepatocellular carcinoma, pancreatic cancer, prostate cancer, head and neck squamous cell carcinoma, ovarian cancer, Epstein-Barr Virus (EBV)-positive advanced malignant tumors, Hepatitis B Virus (HBV)-related refractory hepatocellular carcinoma, glioblastoma, Claudin 6 (CLDN6)-positive relapsed or refractory advanced solid tumors, metastatic neoplasm, relapsed/refractory solid tumor malignancies, and solid tumors cancer [156,157].

Unlike traditional vaccines aimed at preventing infectious diseases, cancer vaccines are primarily therapeutic and designed to treat existing malignancies rather than prevent them [158]. These vaccines contain antigens found on cancer cells, which can be proteins or other molecules that are recognized as foreign by the immune system. Upon administration, the vaccine presents these antigens to dendritic cells, which process and introduce them into the T cells. CD4+ T cells, also known as helper T cells, coordinate the immune response by activating and guiding CD8+ T cells and B cells. CD4+ cells recognize antigens presented by MHC class II molecules, release cytokines that regulate the immune system, and aid CD8+ cells in eliminating tumor cells. CD8+ T cells or cytotoxic T cells directly destroy cancerous cells by recognizing antigens displayed by MHC class I molecules in all body tissues [152].

Cancer vaccines can be either preventive, targeting cancer-causing viruses like human papillomavirus (HPV) to prevent certain cancers, or therapeutic, designed to treat existing cancers by bolstering the body’s natural defenses against cancer cells. The advent of mRNA technology has significantly advanced this field, enabling the rapid design of vaccines that can target specific cancer antigens with high precision [159].

Despite the success of targeted therapies and immunotherapies against various malignancies, responses to these treatments are often inadequate or only transient. A deeper understanding of the impact of targeted treatments on antitumor immunity could lead to more successful combination strategies, resulting in rapid tumor shrinkage and sustained responses [160]. Recent developments aim to enhance cancer therapies by reducing tumor escape rates through better antigen selection, improved immunotherapy delivery, and combination therapy with radiography and chemotherapeutics, all of which have shown synergistic effects with cancer vaccines.

While vaccines targeting human viruses associated with certain cancers, such as human papillomavirus in cervical cancer, are not the primary focus of current studies, cancers produce “altered self” antigens that elicit weaker immune responses compared to foreign antigens from infectious organisms. Recently, immune stimulants and adjuvant approaches have garnered significant interest [161].

Lorentzen et al. highlighted that during the Coronavirus Disease 2019 (COVID-19) pandemic, research on mRNA vaccines as potential cancer treatments accelerated the development of these vaccines. They are simple to produce and have good safety profiles, making them promising agents for cancer immunotherapy. Technological advancements have improved the stability, structure, and dispersion of mRNA vaccines. Early trials reported positive results using mRNA vaccines as monotherapy or in combination with checkpoint inhibitors. Ongoing clinical trials are recruiting individuals with various forms of cancer [154].

Recent studies have explored the use of mRNA vaccines, both as monotherapy and in combination with checkpoint inhibitors in various cancer types. The following are detailed examples.

- mRNA-5671 (Kirsten Rat Sarcoma viral oncogene homolog (KRAS) gene driver mutations) in combination with Pembrolizumab: A Phase 1 clinical trial (NCT03948763) is evaluating the safety and efficacy of the mRNA-5671 vaccine, which targets KRAS mutations in NSCLC, colorectal cancer, and pancreatic cancer. The trial investigates the vaccine both as a monotherapy and in combination with Pembrolizumab, a PD-1 checkpoint inhibitor, highlighting its potential in targeting KRAS-mutated tumors [154].
- mRNA-4157 in Melanoma: Two Phase 1/2 trials (NCT03313778 and NCT03897881) are assessing mRNA-4157, a personalized cancer vaccine encoding several neoantigens. In one study, the vaccine is used as monotherapy in patients with completely resected solid tumors. Another study combines mRNA-4157 with Pembrolizumab in patients with unresectable solid tumors, including melanomas. These trials have shown that the vaccine can induce neoantigen-specific T-cell responses, with some patients experiencing partial or complete responses [154].
- BioNTech111 (BNT111) in Melanoma: BNT111, an mRNA vaccine encoding four melanoma tumor-associated antigens, has been tested in a Phase 1 trial (NCT02410733). This study included both monotherapy and combination therapy with PD-1 inhibitors. The results indicated that BNT111 induced robust CD4+ and CD8+ T-cell responses, and when combined with PD-1 therapy, some patients experienced partial responses [154].
- CARVac (CLDN6) in Solid Tumors: A Phase 1/2 trial (NCT04503278) is exploring the use of CARVac, an mRNA vaccine encoding CLDN6, in combination with CLDN6-targeting CAR-T cell therapy in patients with relapsed or refractory advanced solid tumors. The trial has shown promising results, with partial responses observed in the majority of evaluable patients, indicating the potential of mRNA vaccines to enhance the efficacy of CAR-T cell therapies [154].
- BioNTech111 (BNT122) in Combination Therapies: BNT122, an mRNA vaccine that encodes individual tumor mutations, has been evaluated in several trials (NCT03289962, NCT03815058) for its use in combination with checkpoint inhibitors, such as Atezolizumab and Pembrolizumab. These trials aim to assess the efficacy of combining personalized mRNA vaccines with checkpoint inhibitors in treating advanced melanoma and other solid tumors [154].

The role of immune stimulants and adjuvant approaches in enhancing the efficacy of cancer vaccines is crucial, particularly, given the poor immunogenicity of many cancer antigens. Adjuvants are used to trigger strong and long-lasting immune responses and are typically classified as depot adjuvants (e.g., aluminum hydroxide, emulsions, liposomes) that prolong antigen availability and immunostimulants (e.g., Toll-Like Receptor agonists, saponins, cytokines) [162,163]. These are vital for achieving effective immune responses, especially in older individuals where immunosenescence—a decline in immune function—compromises vaccine efficacy [164,165,166]. Therefore, selecting appropriate adjuvants is critical for overcoming immune tolerance and maximizing efficacy even in compromised environments [163]. Additionally, novel approaches like vector-based antigen delivery and unconventional methods, such as exercise, diet, and psychological care, are being explored to further enhance vaccine effectiveness [167,168].

### 3.6. Cytokine Therapy

Cytokine therapy represents a significant advancement in the field of immunotherapy, offering targeted approaches to modulate the immune system for more effective cancer treatment and the management of immune-mediated diseases. Cytokines, which are small proteins crucial for cell signaling, have been engineered and applied therapeutically to enhance the immune system’s ability to fight cancer and other diseases by manipulating immune responses in a controlled manner [169]. One of the earliest and most well-known applications of cytokine therapy is IL-2, which has been used to stimulate the proliferation and activation of T cells, particularly in the treatment of metastatic renal cell carcinoma and melanoma. High-dose IL-2 therapy has shown significant efficacy, although its use is often limited by severe side effects due to the broad activation of immune cells, including those that may cause systemic inflammation [170].

Advancements in cytokine engineering have led to the development of IL-2 variants, such as the IL-2 “superkine”, which has a higher affinity for the IL-2 receptor on effector T cells and NK cells, enhancing their antitumor activity while reducing the activation of regulatory T cells that could otherwise suppress the immune response [171]. This modification demonstrates how bioengineering can refine cytokine therapy to achieve more targeted and effective outcomes, reduce toxicity, and improve patient tolerance.

Similarly, IL-12, a cytokine known for its role in promoting the differentiation of naive T cells into T helper 1 (Th1) cells and activating NK cells, has been engineered into immunocytokines by fusing it with tumor-targeting antibodies. This fusion enhances the localization of IL-12 to tumor sites, thereby boosting its therapeutic efficacy and minimizing systemic toxicity, which has been a significant challenge in cytokine therapy [172]. These immunocytokines represent a new generation of cytokine therapies that combine the specificity of monoclonal antibodies with the potent immune-stimulating effects of cytokines, offering a more precise treatment option for patients with cancer [173].

Moreover, GM-CSF has been utilized in genetically modified T cells to target specific malignancies, such as those of the central nervous system, illustrating the potential of cytokine therapy to direct immune responses to challenging areas within the body. GM-CSF plays a crucial role in enhancing the recruitment and activation of dendritic cells, which are essential for initiating a robust immune response against tumors [174].

In addition to these approaches, IL-15 has emerged as a key cytokine in enhancing the effectiveness of CAR NK cell therapies. IL-15 not only promotes the survival and proliferation of NK cells but also enhances their cytotoxic activity against cancer cells, making it a valuable component of adoptive cell therapy strategies. Clinical trials have demonstrated that IL-15 can significantly improve the persistence and effectiveness of CAR NK cells in targeting CD19-positive lymphoid tumors, offering new hope for patients with certain types of cancer [175].

The advent of mRNA technology has also revolutionized cytokine therapy by enabling the delivery of cytokines in a more controlled and sustained manner. Lipid nanoparticles encapsulating mRNA encoding for cytokines like IL-12 have shown promising results in preclinical models, where they have effectively suppressed tumor growth with minimal systemic toxicity. This approach not only allows for precise control of cytokine expression but also overcomes some of the pharmacokinetic challenges associated with traditional cytokine therapies [176].

Despite these advancements, challenges remain in the widespread application of cytokine therapy. The potential for cytokine-induced toxicity, particularly with systemic administration, requires ongoing refinement of these therapies to ensure they are both effective and safe for patients. Additionally, the complex regulation of immune responses by cytokines necessitates further research to optimize dosing regimens, delivery methods, and combination strategies with other immunotherapies such as checkpoint inhibitors [177]. As research continues, cytokine therapy holds immense potential as a cornerstone of modern cancer treatment and beyond.

### 3.7. Immunomodulators 

Immunomodulators play an increasingly pivotal role in cancer therapy by enhancing the body’s natural immune responses to target and eliminate tumor cells. These agents modulate the tumor microenvironment, which is composed of various immune and non-immune cells, to overcome immune evasion strategies employed by cancer cells. Mechanistically, immunomodulators operate through several pathways and cellular interactions that collectively boost the antitumor immune response. One of the key mechanisms involves the activation and enhancement of cytotoxic T cells (CTLs) and DCs, which are essential for initiating and sustaining an effective immune attack on tumors. CTLs, once activated by antigen-presenting cells such as DCs, can directly kill cancer cells. However, in many cancers, these CTLs become exhausted due to the expression of immune checkpoint molecules like PD-1 on their surface and PD-L1 on cancer cells. Immunomodulators can disrupt this interaction, as demonstrated by resveratrol, which targets PD-L1 and prevents its binding to PD-1, thereby restoring CTL activity and promoting tumor cell death [178,179].

Another significant mechanism by which immunomodulators enhance cancer immunity is through the reduction of Tregs and tumor-associated macrophages (TAMs), both of which contribute to an immunosuppressive environment that protects tumors from the immune system. Tregs suppress the activity of conventional T cells, and their abundance in the tumor microenvironment is often correlated with poor prognosis. Immunomodulatory agents like resveratrol have been shown to reduce Treg populations and inhibit their function, thereby enhancing the overall immune response against the tumor. Similarly, TAMs, particularly those of the M2 phenotype, promote tumor growth and metastasis by supporting angiogenesis and suppressing antitumor immunity. Immunomodulators can repolarize TAMs from a tumor-promoting M2 phenotype to a tumor-suppressing M1 phenotype, which enhances the immune system’s ability to attack the cancer. In liver cancer models, for example, resveratrol was observed to decrease the levels of immunosuppressive cytokines like Transforming Growth Factor Beta (TGF-β) and IL-10, while increasing pro-inflammatory cytokines, such as TNF-α and IFN-γ, creating a more hostile environment for the tumor [179,180].

Furthermore, NK cells, which are another critical component of the innate immune system, can be activated by immunomodulators to enhance their cytotoxic activity against cancer cells. NK cells play a vital role in controlling metastasis and the spread of cancer, and their activity is often suppressed in the tumor microenvironment. Agents like resveratrol have been shown to activate NK cells through the Protein Kinase B (also known as AKT)/Mechanistic Target of Rapamycin Complex 2 (mTORC2) signaling pathway, leading to increased expression of NK cell receptors such as Natural Killer Cell p30-Related Protein (NKp30) and Natural Killer Group 2, Member D (NKG2D), which are crucial for recognizing and killing cancer cells. Additionally, resveratrol enhances the expression of MHC Class I Polypeptide-Related Sequence A (MICA) and MHC Class I Polypeptide-Related Sequence B (MICB) in cancer cells, making them more susceptible to NK cell-mediated lysis. These mechanisms collectively highlight the potential of immunomodulators in reversing the immunosuppressive nature of the tumor microenvironment and improving the efficacy of cancer immunotherapy. Their ability to enhance the activity of various immune cells, reduce immunosuppressive factors, and modulate key signaling pathways underscores their promise as adjuncts to existing cancer treatments, offering new avenues for more effective and durable therapeutic outcomes [179,181].

## 4. Conclusions

Cancer immunotherapy has revolutionized oncology by harnessing the immune system’s ability to combat malignancies with greater precision and reduced toxicity compared to traditional therapies. Significant advancements in monoclonal antibodies, CAR-T cell therapy, checkpoint inhibitors, and cancer vaccines have demonstrated substantial efficacy in various cancer types. However, as the field continues to evolve, several key challenges and opportunities must be addressed. Table 1 summarizes the key components and impacts of this review on cancer immunotherapy.

### 4.1. Future Directions in Cancer Immunotherapy Research

Future research on cancer immunotherapy is expected to focus on overcoming immune resistance and improving treatment durability. Emerging technologies, such as next-generation antibodies, personalized cancer vaccines, and advanced CAR-T cell therapies, have the potential to address the limitations of the current therapies. For instance, the development of bispecific antibodies and antibody-drug conjugates offers new strategies for targeting cancer cells more effectively. The integration of mRNA technology, which has shown promise in recent trials, is likely to further advance the field by enabling rapid and precise vaccine development tailored to individual patients.

### 4.2. Challenges in Cancer Immunotherapy

Despite these advancements, significant challenges remain, particularly regarding immune resistance and the durability of treatment responses. Tumor heterogeneity, immune evasion, and the immunosuppressive tumor microenvironment continue to pose obstacles to the long-term success of immunotherapy. Additionally, the specificity of tumor antigens and the need for sustained immune activation without causing autoimmunity are critical areas that require further investigation.

## Figures and Tables

**Table 1:** Summarizes the key components and impacts of this review on cancer immunotherapy.

Category	Details	Impact
Monoclonal Antibodies	- Examples: Rituximab (CD20), Trastuzumab (HER2), Bevacizumab (VEGF)	- Revolutionized treatment for cancers like lymphoma and breast cancer.
	- Mechanisms: Growth factor signaling inhibition, ADCC, CDC	- Ongoing need for combination therapies to overcome resistance and improve long-term outcomes.
CAR-T Cell Therapy	- Advances: Second and third-generation CAR-T cells, fourth-generation “armored” CAR-T cells	- Offers potentially curative options for refractory hematologic cancers.
	- Applications: Hematologic malignancies, emerging use in solid tumors	- Requires further research for effective application in solid tumors and overcoming TME barriers.
Checkpoint Inhibitors	- Key inhibitors: PD-1 (Pembrolizumab, Nivolumab), PD-L1 (Atezolizumab), CTLA-4 (Ipilimumab)	- Transformative for cancers like melanoma and lung cancer.
	- Mechanisms: Restoring T cell function, blocking immune suppression	- Necessitates management of immune-related adverse events and strategies to sustain responses.
Cancer Vaccines	- Technologies: mRNA vaccines (e.g., BNT111, mRNA-4157), personalized neoantigen vaccines	- Represents a frontier in personalized cancer treatment.
	- Applications: Solid tumors, in combination with checkpoint inhibitors	- Promising in clinical trials but requires further refinement for broader efficacy.
Cytokine Therapy	- Key cytokines: IL-2, IL-12, GM-CSF	- Critical in boosting immune responses in cancer therapy.
	- Mechanisms: Enhancing T cell and NK cell activity, modulating the tumor microenvironment	- Advances in engineering cytokines offer more targeted and safer therapeutic options.
Next-Generation Antibodies	- Innovations: Bispecific antibodies, Fc-engineered antibodies, ADCs	- Paves the way for more precise and effective cancer therapies.
	- Applications: Enhanced targeting, improved safety profiles	- Ongoing development is needed to translate laboratory advancements into clinical success.
Immunomodulators	- Focus: Modifying TME, reducing Tregs and TAMs, activating NK cells	- Supports the enhancement of existing immunotherapy regimens.
	- Mechanisms: Targeting PD-L1, promoting a pro-inflammatory environment	- Requires careful balancing of immune activation with the potential for adverse reactions.
Emerging Technologies	- Advances: CRISPR-based gene editing, AI-driven drug discovery, mRNA delivery systems	- Poised to significantly advance the field of cancer immunotherapy.
	- Applications: Targeted cancer vaccines, CAR-T cell modifications	- Critical for overcoming existing limitations in specificity, safety, and efficacy.
